# Recent advancements on the phase field approach to brittle fracture for heterogeneous materials and structures

**DOI:** 10.1186/s40323-018-0102-y

**Published:** 2018-05-02

**Authors:** V. Carollo, T. Guillén-Hernández, J. Reinoso, M. Paggi

**Affiliations:** 1 IMT School for Advanced Studies Lucca, Piazza San Francesco 19, 55100 Lucca, Italy; 2 Elasticity and Strength of Materials Group, School of Engineering, Universidad de Sevilla, Camino de los Descubrimientos s/n, 41092 Seville, Spain

**Keywords:** Phase field model of fracture, Cohesive zone model, Solid shell, Heterogeneous materials, Nonlinear finite element method

## Abstract

Recent advancements on the variational approach to fracture for the prediction of complex crack patterns in heterogeneous materials and composite structures is herein proposed, as a result of the frontier research activities undertaken in the FP7 ERC Starting Grant project CA2PVM which focuses on the development of computational methods for the durability and the reliability assessment of photovoltaic laminates. From the methodological viewpoint, the phase field approach to describe the propagation of brittle fracture in the bulk has been coupled for the very first time with the cohesive zone model to depict interface crack growth events, for 2D isotropic and anisotropic constitutive laws, and also for 3D finite elasticity. After a summary of the key aspects underlying the theoretical formulation and the finite element implementation using a monolithic fully implicit solution scheme, an overview of the main technological applications involving layered shells, interface mechanical problems and polycrystalline materials is provided. The examples are selected to show the capability of the proposed approach to investigate complex phenomena such as crack deflection vs. crack penetration at an interface, intergranular vs. transgranular crack growth in polycrystals, and interlayer vs. translayer failure in laminates.

## Introduction

The phenomenology and modelling of brittle fracture is a well established topic in the area of fracture mechanics. In the last decades, many computational models have been developed in order to predict the strength and the crack path in brittle materials. Techniques such as the extended finite element method (XFEM) [1, 2], finite fracture mechanics (FFM) [3] and the embedded finite element method (EFEM) [4] belong to this class of approaches. In parallel, methods to simulate quasi-brittle and interface fracture adopting the cohesive zone model (CZM) have been proposed to simulate cohesive fracture typical of elasto-plastic materials or interfaces. These computational models generally suffer from some drawbacks due to the complexity in tracing the evolution of the crack path. On the one side in XFEM and EFEM, a splitting algorithm should be defined for the element crossed by the crack or containing the crack tip. This leads to crack topology problems when the quadratic or higher order displacement interpolation schemes are employed. This is not the case of FFM which is a powerful and well-founded predictive tool, but can only by applied to relatively simple structures for feasibility reasons. On the other side, CZM-based methods, implemented using interface finite elements, are viable strategies for pre-existing interfaces [5, 6], while several algorithmic complexities arise for modelling evolving cracks in the continuum [7, 8].

To circumvent these drawbacks, regularization schemes based on non-local gradient damage formulations have been developed in the last decades [9, 10] whereby some of these proposals incorporated the use of an additional equation in which the length-scale was explicitly considered [11–13]. In this regard, the phase field method for fracture has been proposed and enhanced by many authors [14–16] and subsequently assessing its thermodynamic consistency in [17, 18]. The phase field method considers the crack as a diffuse damage instead of a sharp discontinuity. This regularization is formulated through a variational approach of the classical Griffith energy balance for brittle fracture. The main advantage of this method is that the phase-field/damage variable is considered as an additional primary unknown of the problem to be determined along the solution process, i.e. generally leading to the definition of an extra degree of freedom per node in the corresponding FE discretization. Consequently, crack nucleation as well as the crack path are predicted as a result of an energy minimization problem without the necessity of a remeshing algorithm to treat the evolution of damage.

In the present review article, we focus on the recent advancements in the simulations of crack growth in heterogeneous materials and composite structures, motivated by the frontier research undertaken in the ERC Starting Grant “Multi-field and Multi-scale Computational Approach to Design and Durability of PhotoVoltaic Modules” (CA2PVM). The prediction of the reliability and durability of photovoltaic laminates subject to mechanical loads requested in fact the development of advanced computational models to simulate delamination in multi-layered composites and, at the same time, new fracture mechanics approaches to predict fracture in polycrystalline silicon solar cells, which is concern in photovoltaics due to its brittleness [19]. Global–local approaches in order to bridge the scales between fracture at the scale of the laminate and crack growth at the level of the solar cell have also been proposed in [20, 21] with a novel multi-field perspective.

Due to its versatility and capability of the proposed approach to simulate complex crack paths, we first recall the fundamental features of the phase field formulation for fracture in “Foundation of the phase field method” section. Then, in “Advancements on the phase field method: formulation and finite element implementation” section, the main theoretical and computational advancements are presented. Finally, “Applications to heterogeneous materials and composite structures” section is devoted to selected applications where complex fracture phenomena in heterogeneous materials and structures are predicted.

## Foundation of the phase field method

In this section we describe the thermodynamically consistent formulation of the phase field method for brittle fracture proposed in [17, 22]. This formulation lays in the classical fracture theory of Griffith, but it considers the crack as a diffuse damage instead of a sharp discontinuity.

**Fig. 1:**
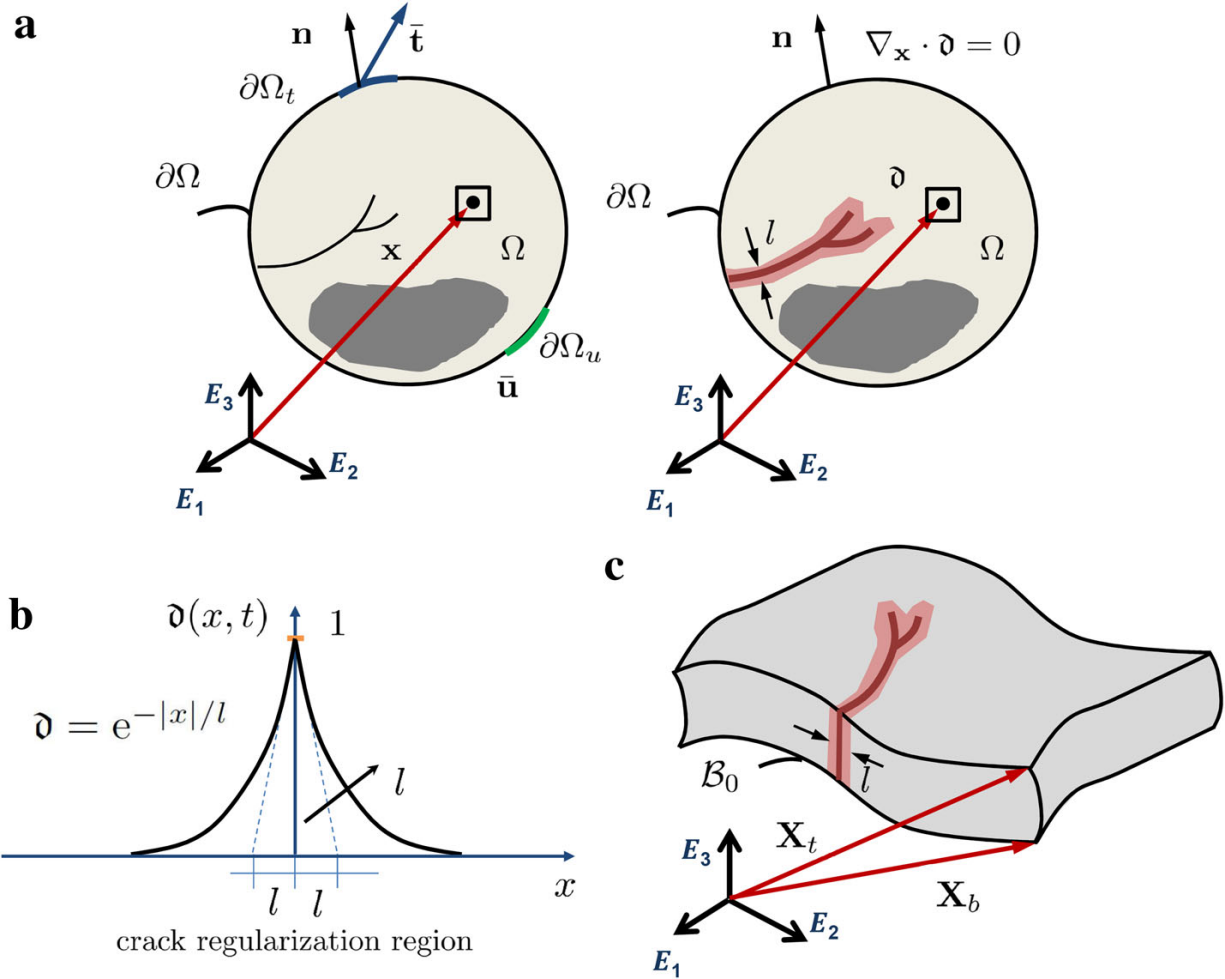
**a** Comparison between the discrete discontinuity of the LEFM theory (left) with the smeared discontinuity of the PF model (right); **b** 1D approximation function which smear out the discontinuity, the damage ${\mathfrak {d}}$ follows the exponential based function ${\mathfrak {d}} = \text {e}^{-\mid x \mid / l}$. **c** Phase field approach for thin-walled structures adopting the solid shell approach

The approach is developed in the general multi-dimensional framework (Fig. 1a). A deformable body $\Omega \in {\mathbb {R}}^n$ in the Euclidean space is considered. An arbitrary point in the body $\Omega$ is defined by the vector of its Cartesian coordinates $\mathbf x$, while the body forces are denoted by $\mathbf{f }_\mathbf{v }: \Omega \longrightarrow {\mathbb {R}}^n$. The boundaries of $\Omega$ are denoted by $\partial \Omega \in {\mathbb {R}}^{n-1}$, which are split into the prescribed kinematic boundary $\partial \Omega _\text {u}$ and the prescribed traction boundary $\partial \Omega _\text {t}$. Kinematic and traction boundaries fulfill the two conditions: $\partial \Omega _{t}\cup \partial \Omega _{u}=\partial \Omega$ and $\partial \Omega _{t}\cap \partial \Omega _{u}=\emptyset$. For a generic point of $\Omega$, the displacement vector is denoted by $\mathbf u$ and the Cauchy stress tensor by ${\varvec{\sigma }}$. Then, the prescribed displacements and tractions at the respective boundaries are denoted by:

1 $$\begin{aligned} \mathbf {u} = \mathbf {\overline{u}} \,\text { on } \partial \Omega _{u} \, \text { and } \, \mathbf {\overline{t}} = {\varvec{\sigma }} \cdot \mathbf {n} \, \text { on } \partial \Omega _{t}, \end{aligned}$$

where $\mathbf n$ denotes the outward normal unit vector to the body.

In the phase field model for brittle fracture, the crack, which is usually represented by a discrete discontinuity, is regularized through the diffuse phase field damage variable ${\mathfrak {d}}$, with ${\mathfrak {d}} : \Omega$ x $[0,t] \longrightarrow [0,1]$ [16]. The value ${\mathfrak {d}}=0$ denotes the undamaged state, while ${\mathfrak {d}}=1$ identifies the fully damaged state. The phase field damage variable can vary from 0 to 1 as shown in Fig. 1b, with the closed-form expression ${\mathfrak {d}}=\text {e}^{-\mid x \mid / l}$ available only for the simple mono-dimensional problem. For higher dimensions, a closed-form expression is not available and implicitly depends on the phase field internal length scale *l*, which determines the band where damage spreads [17, 22].

According to this framework, the potential energy of the system takes the form:

2 $$\begin{aligned} \Pi (\mathbf {u}, {\mathfrak {d}}) = \int _{\Omega } \psi ({\varvec{\varepsilon }},{\mathfrak {d}}) \,\mathrm {d}\Omega +\int _\Omega {\mathcal {G}}_{c} \gamma ({\mathfrak {d}}, \nabla _{\mathbf {x}} {\mathfrak {d}}) \,\mathrm {d}\Omega \text {,} \end{aligned}$$

where $\psi ({\varvec{\varepsilon }},{\mathfrak {d}})$ is the elastic energy density which depend on the damage ${\mathfrak {d}}$ and the strain ${\varvec{\varepsilon }}$. Here, ${\mathcal {G}}_{c}$ is the Griffith fracture energy and $\gamma ({\mathfrak {d}}, \nabla _{\mathbf {x}} {\mathfrak {d}})$ is the so-called crack density functional which depends on ${\mathfrak {d}}$ and its gradient $\nabla _{\mathbf {x}} {\mathfrak {d}}$. The crack density functional is defined in [17] as:

3 $$\begin{aligned} \gamma ({\mathfrak {d}}, \nabla _{\mathbf {x}} {\mathfrak {d}}) = \frac{1}{2l} {\mathfrak {d}}^{2} + \dfrac{l}{2} |\nabla _{\mathbf {x}} {\mathfrak {d}} |^{2}. \end{aligned}$$

The Euler equations (strong form) associated with the phase field variable read:

4 $$\begin{aligned} {\mathfrak {d}} - l^{2} \nabla _{\mathbf {x}}^{2} {\mathfrak {d}} = 0 \, \text { in }\Omega \, \text { and } \, \nabla _{\mathbf {x}} {\mathfrak {d}} \cdot \mathbf {n} = 0 \, \text { in }\partial \Omega , \end{aligned}$$

where $\nabla _{\mathbf {x}}^{2} {\mathfrak {d}}$ is the Laplacian of the phase field variable.

Regarding the elastic energy stored in the body $\psi ({\varvec{\varepsilon }},{\mathfrak {d}})$, the positive–negative split based on a spectral decomposition of the strain tensor is usually adopted following [23]. The positive counterpart of the elastic energy depends on the tensile stresses, while the negative counterpart depends on the compressive stresses. Following [17], the positive–negative split takes the form:

5a $$\begin{aligned} \psi ({\varvec{\varepsilon }},{\mathfrak {d}})&= {\mathfrak {g}}({\mathfrak {d}}) \psi ^{e}_{+}({\varvec{\varepsilon }}) + \psi ^{e}_{-}({\varvec{\varepsilon }}), \end{aligned}$$

5b $$\begin{aligned} \psi ^{e}_{+}({\varvec{\varepsilon }})&= \dfrac{\lambda }{2} \left( \langle \text {tr} [{\varvec{\varepsilon }} ] \rangle _{+ } \right) ^{2} + \mu \text {tr} [{\varvec{\varepsilon }}_{+}^{2} ], \end{aligned}$$

5c $$\begin{aligned} \psi ^{e}_{-}({\varvec{\varepsilon }})&= \dfrac{\lambda }{2} \left( \langle \text {tr} [{\varvec{\varepsilon }} ] \rangle _{- } \right) ^{2} + \mu \text {tr} [{\varvec{\varepsilon }}_{-}^{2} ], \end{aligned}$$

where $\lambda$ and $\mu$ are the Lamé constants, $\varepsilon _{+}$ and $\varepsilon _{-}$ are, respectively, the positive and negative counterparts of the strain tensor. The symbol $\text {tr} [\bullet ]$ denotes the trace operator, and the symbol $\langle \bullet \rangle _{\pm }$ is the so-called Macaulay brackets corresponding to the function $\langle \bullet \rangle _{\pm } = (\bullet \pm | \bullet |)/2$. The function ${\mathfrak {g}}({\mathfrak {d}})$ is a degradation function that, in the classical formulation, takes the form:

6 $$\begin{aligned} {\mathfrak {g}}({\mathfrak {d}}) = \left( 1 - {\mathfrak {d}} \right) ^{2} + \mathcal {K}, \end{aligned}$$

where $\mathcal {K}$ is a residual stiffness which is introduced to avoid numerical instabilities and an ill-conditioned stiffness matrix when ${\mathfrak {d}}$ approaches unity. In Eq. (5a) the degradation function (Eq. 6) multiplies only the positive part of the elastic energy. In this way, damage affects only the portion of the body where tensile stresses are developed, avoiding fracture in compression. Hence, the Cauchy stress tensor of the phase field formulation takes the form:

7 $$\begin{aligned} {\varvec{\sigma }} : = \frac{\partial \hat{\psi }}{\partial {\varvec{\varepsilon }}} = {\mathfrak {g}}({\mathfrak {d}}) {\varvec{\sigma }}_{+} + {\varvec{\sigma }}_{-}; \,\, \text { with } {\varvec{\sigma }}_{\pm } = \lambda \left( \langle \text {tr} [{\varvec{\varepsilon }} ] \rangle _{\pm } \right) \mathbf {1} + 2 \mu {\varvec{\varepsilon }}_{\pm }, \end{aligned}$$

where $\mathbf {1}$ denotes the second-order identity tensor, and $\sigma _{\pm }$ denotes the positive–negative counterpart of the stress tensor.

## Advancements on the phase field method: formulation and finite element implementation

In this section the main advancements of the phase field model to provide computational tools for the simulation of complex crack paths are reviewed.

In the sequel, we first present the extension of the 2D phase field model to a 3D finite elasticity solid shell finite element (“Fracture in solid shells with finite elasticity” section). Subsequently, in “Coupling the phase field for brittle fracture and the cohesive zone model for interface debonding” section, we present the formulation and finite element implementation of a novel interface element compatible with the phase field model. Finally, the enhancement of this interface element to a 3D finite elasticity framework is outlined in “Interlaminar and translaminar fracture in laminates combining the 3D approach to brittle fracture for solid shells and a finite elasticity interface finite element for decohesion” section.

### Fracture in solid shells with finite elasticity

#### Phase field formulation for solid shells relying on the enhanced assumed strain (EAS) method

The development of numerical methods to trigger fracture events in thin-walled structures has been a recurrent topic in the last three decades. Within this context, in [24], the phase field approach to fracture has been reformulated in order to be used in thin walled applications recalling the solid shell finite concept (Fig. 1c). In the current solid shell formulation, the enhanced assumed strain (EAS) technology has been used to alleviate locking pathologies. In particular, the EAS method has been advocated through the adoption of the additive decomposition of the Green–Lagrange strain tensor $\mathbf {E} = \mathbf {E}^{u} + \mathbf {\tilde{E}}$, where $\mathbf {E}^{u}$ and $\mathbf {\tilde{E}}$ denote the compatible and the incompatible counterparts of the strain [25, 26], respectively.

The formulation is defined through the exploitation of the Hu–Washizu functional. The displacements $\mathbf {u}$, the incompatible strains $\tilde{\mathbf {E}}$, the second Piola–Kirchhoff stress tensor $\mathbf {S}$, and the crack phase field variable ${\mathfrak {d}}$ constitute the independent fields of the formulation. According to the Griffith’s theory of brittle fracture, for cracked bodies, this variational formalism in the reference configuration can be expressed as:

8 $$\begin{aligned} \Pi (\mathbf {u}, \mathbf {\tilde{E}}, \mathbf {S}, {\mathfrak {d}}) = \underbrace{\int _{{\mathcal {B}}_{0} \backslash \Gamma } {\mathfrak {g}}({\mathfrak {d}}) \Psi ( \mathbf {{E}})\,{\mathrm {d}}\Omega - \int _{{\mathcal {B}}_{0}} \mathbf {S}: \mathbf {\tilde{E}} \,{\mathrm {d}}\Omega }_{\Pi _{\text {int}}^{b}} + \underbrace{\int _{\Gamma } {\mathcal {G}}_{c} \,{\mathrm {d}}\Gamma }_{\Pi _{\text {fr}}} + \Pi _{\text {ext}}, \end{aligned}$$

where the internal contribution of the bulk is denoted by $\Pi _{\text {int}}^{b}$, whilst $\Pi _{\text {fr}}$ identifies the dissipative contribution due to fracture events. Furthermore, the prescribed external surface and body actions are arranged in the term $\Pi _{\text {ext}}$. In Eq. (8), $\Psi (\mathbf {E})$ is the effective Helmholtz free-energy function in the bulk for undamaged hyperelastic materials and ${\mathfrak {g}}({\mathfrak {d}})$ is the degradation function (Eq. 6). Moreover, it is worth mentioning that the second integral term of $\Pi _{\text {int}}^{b}$ accounts for the contribution of the second Piola–Kirchhoff stress tensor ($\mathbf {S}$) over the incompatible strains ($\mathbf {\tilde{E}}$) in the view of the EAS method.

Introducing the phase field approximation to the dissipated energy counterpart of the functional in Eq. (8), it follows:

9 $$\begin{aligned} \Pi (\mathbf {u}, \mathbf {\tilde{E}}, \mathbf {S}, {\mathfrak {d}}) = \int _{{\mathcal {B}}_{0} \backslash \Gamma } {\mathfrak {g}}({\mathfrak {d}}) \Psi ( \mathbf {{E}})\,{\mathrm {d}}\Omega - \int _{{\mathcal {B}}_{0}} \mathbf {S}: \mathbf {\tilde{E}} \,{\mathrm {d}}\Omega + \int _{{\mathcal {B}}_{0}} {\mathcal {G}}^{b}_{c} \gamma ({\mathfrak {d}}, \nabla _{\mathbf {X}} {\mathfrak {d}}) \,{\mathrm {d}}\Omega + \Pi _{\text {ext}} \end{aligned}$$

The corresponding weak form of the functional in Eq. (9) is given by the first variation with respect to the four independent fields, and by the imposition of the orthogonality condition for the stress field $\mathbf {S}$ and the enhanced strain field $\mathbf {\tilde{E}}$ [27]. Then, the weak form of the boundary value problem associated with the cracked bulk is reduced to the following three-field problem:

10 $$\begin{aligned}&\mathcal {R}^{u} ( \mathbf {u}, \delta \mathbf {u}, \mathbf {\tilde{E}}, {\mathfrak {d}} ) = \mathcal {R}^{u}_{\text {int}} - \mathcal {R}^{u}_{\text {ext}} = \int _{{\mathcal {B}}_{0}} {\mathfrak {g}}({\mathfrak {d}}) \frac{\partial \Psi }{\partial \mathbf {E}} : \delta \mathbf {E}^{u} \,{\mathrm {d}}\Omega + \delta \Pi _{\text {ext}} (\mathbf {u}) = 0 , \end{aligned}$$

11 $$\begin{aligned}&\mathcal {R}^{\tilde{E}} ( \mathbf {u}, \mathbf {\tilde{E}}, \delta \mathbf {\tilde{E}}, {\mathfrak {d}} ) = \int _{{\mathcal {B}}_{0}} {\mathfrak {g}}({\mathfrak {d}}) \frac{\partial \Psi }{\partial \mathbf {E}} : \delta \tilde{\mathbf {E}} \,{\mathrm {d}}\Omega = 0, \end{aligned}$$

12 $$\begin{aligned}&\mathcal {R}^{{\mathfrak {d}}} ( \mathbf {u}, \mathbf {\tilde{E}}, {\mathfrak {d}}, \delta {\mathfrak {d}} ) = \int _{{\mathcal {B}}_{0}} -2(1- {\mathfrak {d}}) \delta {\mathfrak {d}} \Psi ( \mathbf {E}) \,{\mathrm {d}}\Omega \nonumber \\&\quad \quad \quad \quad \quad \quad \quad \quad + \int _{{\mathcal {B}}_{0}} {\mathcal {G}}^{b}_{c}l\left[ \frac{1}{l^{2}} {\mathfrak {d}} \delta {\mathfrak {d}} + \nabla _{\mathbf {X}} {\mathfrak {d}} \cdot \nabla _{\mathbf {X}} (\delta {\mathfrak {d}}) \right] \,{\mathrm {d}}\Omega = 0. \end{aligned}$$

The nonlinear system given by Eqs. (10)–(12) can be solved by means of the standard incremental-iterative Newton–Raphson method. The linearization leads to a fully coupled system of equation, which has been tackled using a monolithic solution strategy [24].

Regarding the material formulation, a hyperelastic neo-Hookean isotropic constitutive response has been assumed for the numerical implementation of the current framework, with:

13a $$\begin{aligned} \Psi (\mathbf {C})&= \frac{\lambda }{2} \left( \text {ln} J \right) ^{2} - \mu \text {ln} J + \frac{\mu }{2} \left( \text {tr}[\mathbf {C}] - 3 \right) , \end{aligned}$$

13b $$\begin{aligned} \mathbb {C}(\mathbf {C})&= 4 \partial _{\mathbf {C} \mathbf {C}} \Psi (\mathbf {C}) = \lambda \mathbf {C}^{-1} \otimes \mathbf {C}^{-1} + 2\left( \lambda \text {ln} J - \mu \right) \frac{\partial \mathbf {C}^{-1}}{\partial \mathbf {C}}, \end{aligned}$$

where *J* identifies the determinant of the deformation gradient $\mathbf {F}$ accounting for the consideration of the incompatible strains [24].

Finally, standard Dirichlet-type and Neumann-type boundary conditions should be considered for the boundary value problem in the bulk. Standard discretization of the domain ${\mathcal {B}}_{0}$ is considered to be constructed via $n_{e}$ non-overlapping elements, such that ${\mathcal {B}}_{0} \approx \bigcup _{e=1} ^{n_{e}} {\mathcal {B}}_{0}^{(e)}$. The discretization of the bulk is performed according to the solid shell concept [28, 29], being the parametric space identified as follows: $\mathcal {A} : = \{ {\varvec{\xi }} = (\xi ^{1},\xi ^{2},\xi ^{3}) \in {\mathbb {R}}^{3} | -1 \le \xi ^{i} \le +1; i =1,2,3 \}$, where ($\xi ^{1},\xi ^{2}$) denote in-plane directions, whereas $\xi ^{3}$ identifies the thickness direction and *H* is the initial shell thickness.

The reference position vector of any material point is linearly interpolated by the position of the top $\mathbf X _{t}(\xi ^{1},\xi ^{2})$ and bottom $\mathbf X _{b}(\xi ^{1},\xi ^{2})$ vectors:

14 $$\begin{aligned} \mathbf X ({\varvec{\xi }}) = \frac{1}{2}\left( 1 + \xi ^{3}\right) \mathbf X _{t}(\xi ^{1},\xi ^{2}) + \frac{1}{2}\left( 1 - \xi ^{3}\right) \mathbf X _{b}(\xi ^{1},\xi ^{2}). \end{aligned}$$

Similarly, the same interpolation scheme is adopted for the current configuration:

15 $$\begin{aligned} \mathbf x ({\varvec{\xi }}) = \frac{1}{2}\left( 1 + \xi ^{3}\right) \mathbf x _{t}(\xi ^{1},\xi ^{2}) + \frac{1}{2}\left( 1 - \xi ^{3}\right) \mathbf x _{b}(\xi ^{1},\xi ^{2}). \end{aligned}$$

The previous approximation is also assumed for the phase field variable:

16 $$\begin{aligned} {\mathfrak {d}}({\varvec{\xi }}) = \frac{1}{2}\left( 1 + \xi ^{3}\right) {\mathfrak {d}}_{t}(\xi ^{1},\xi ^{2}) + \frac{1}{2}\left( 1 - \xi ^{3}\right) {\mathfrak {d}}_{b}(\xi ^{1},\xi ^{2}), \end{aligned}$$

where ${\mathfrak {d}}_{t}$ and ${\mathfrak {d}}_{b}$ stand for the phase field variable values corresponding to the top and bottom surfaces of the body, respectively. This ansazt for the phase field variable allows a non-uniform value of this parameter over the shell thickness, an important result as discussed in [24].

Standard tri-linear shape functions are used to interpolate the reference and current position vectors:

17 $$\begin{aligned} \mathbf {X} = \mathbf {N}({\varvec{\xi }}) \widetilde{\mathbf{X }} \, \text { and } \, \mathbf {x} = \mathbf {N}({\varvec{\xi }}) \widetilde{\mathbf{x }}, \end{aligned}$$

where $\mathbf {N}({\varvec{\xi }})$ is the matrix operator associated with the shape functions.

Accordingly, the displacement and the phase field variable ($\mathbf {u}, {\mathfrak {d}}$), their respective variations ($\delta \mathbf {u}, \delta {\mathfrak {d}}$) and their increments ($\Delta \mathbf {u}, \Delta {\mathfrak {d}}$) are approximated at the element level as:

18 $$\begin{aligned}&\mathbf {u} \approx \mathbf {N}({\varvec{\xi }}) \mathbf {d}; \, \delta \mathbf {u} \approx \mathbf {N}({\varvec{\xi }}) \delta \mathbf {d}; \, \Delta \mathbf {u} \approx \mathbf {N}({\varvec{\xi }}) \Delta \mathbf {d} , \end{aligned}$$

19 $$\begin{aligned}&{\mathfrak {d}} \approx \mathbf {N}({\varvec{\xi }}) \mathfrak {\overline{d}}; \, \delta {\mathfrak {d}} \approx \mathbf {N} ({\varvec{\xi }}) \delta \mathfrak {\overline{d}}; \, \Delta {\mathfrak {d}} \approx \mathbf {N} ({\varvec{\xi }}) \Delta \mathfrak {\overline{d}}. \end{aligned}$$

The interpolation of the incompatible strain field is expressed in terms of the operator $\mathbf {M}({\varvec{\xi }})$ [30] that is designed to alleviate membrane and Poisson thickness locking pathologies through the EAS method. The interpolation of the incompatible strains $\tilde{\mathbf {E}}$, its variation $\delta \tilde{\mathbf {E}}$ and its increment $\Delta \tilde{\mathbf {E}}$ renders

20 $$\begin{aligned} \mathbf {\tilde{E}} \approx \mathbf {M}({\varvec{\xi }}) {\varvec{\varsigma }}, \, \delta \mathbf {\tilde{E}} \approx \mathbf {M}({\varvec{\xi }}) \delta {\varvec{\varsigma }}, \, \Delta \mathbf {\tilde{E}} \approx \mathbf {M}({\varvec{\xi }}) \Delta {\varvec{\varsigma }} . \end{aligned}$$

As discussed in [25, 31], the operator $\mathbf {\tilde{M}}({\varvec{\xi }})$ is subsequently transformed into the global Cartesian setting in order to preserve the consistency of the formulation. Furthermore, transverse shear and trapezoidal locking are circumvented through the use of the assumed natural strain method as detailed in [24].

Insertion of the previous discretization schemes into the residual forms given in Eqs. (10)–(12), and into the corresponding linearized system of equations, leads to the following coupled system:

21 $$\begin{aligned} \begin{bmatrix} \mathbf {k}_{dd}&\mathbf {k}_{d {\mathfrak {d}} }&\mathbf {k}_{{d} \varsigma } \\ \mathbf {k}_{{\mathfrak {d}} d}&\mathbf {k}_{{\mathfrak {d}} {\mathfrak {d}} }&\mathbf {k}_{{\mathfrak {d}} \varsigma } \\ \mathbf {k}_{\varsigma d}&\mathbf {k}_{\varsigma {\mathfrak {d}}}&\mathbf {k}_{\varsigma \varsigma } \end{bmatrix} \begin{bmatrix} \Delta \mathbf {d} \\ \Delta \mathfrak {\overline{d}} \\ \Delta \varvec{\varsigma } \end{bmatrix} = \begin{bmatrix} \mathbf {R}^{d}_{\text {ext}} \\ \varvec{0} \\ \varvec{0} \end{bmatrix} - \begin{bmatrix} \mathbf {R}^{d}_{\text {int}} \\ \mathbf {R}^{ {\mathfrak {d}}}_{\text {int}} \\ \mathbf {R}^{ \varsigma }_{\text {int}} \end{bmatrix} \end{aligned}$$

which can be reduced via the static condensation of the incompatible strains at the element level [24], so that the final system of equations featuring the coupled scheme between the kinematic and the phase field reads:

22 $$\begin{aligned} \begin{bmatrix} \mathbf {k}^{*}_{dd}&\mathbf {k}^{*}_{d {\mathfrak {d}} } \\ \mathbf {k}^{*}_{{\mathfrak {d}} d}&\mathbf {k}^{*}_{{\mathfrak {d}} {\mathfrak {d}} } \end{bmatrix} \begin{bmatrix} \Delta \mathbf {d} \\ \Delta \mathfrak {\overline{d}} \end{bmatrix} = \begin{bmatrix} \mathbf {R}^{d}_{\text {ext}} \\ \varvec{0} \end{bmatrix} - \begin{bmatrix} \mathbf {R}^{d*}_{\text {int}} \\ \mathbf {R}_{\text {int}}^{{\mathfrak {d}}*} \end{bmatrix} \end{aligned}$$

In Eqs. (21) and (22) the operators $k_{mn}$ denote the tangent matrices with respect of the fields *m* and *n*, whilst $R_{ext}^{m}$ and $R_{int}^{m}$ stand for the external and internal residual vectors associated with the field *m*, respectively.

#### Alternative shell models including phase field capabilities for fracture 

The development of shell models incorporating phase field fracture capabilities has received a notable attention in the last few years. In this regard, the formulation outlined in “Phase field formulation for solid shells relying on the enhanced assumed strain (EAS) method” section presented different novel aspects with respect to alternative formulations, which are discussed in the sequel.

One of the pioneering shell formulations triggering fracture events through the phase field approach was developed by Miehe et al. [32], who exploited the use of the standard Kirchhoff (usually denominated as 3-parameter formulation) shell model and restricted to geometrically linear analyses. Moreover, differing from solid shell formulations, this 3-p shell was formulated within the stress–strain resultant spaces leading to the definition of 3 kinematic degrees of freedom per node, which were split into membrane and deflection displacements, and therefore the transverse shear strains along the thickness shell coordinate were neglected. Accordingly, the global energy storage functional for linear elastic solids reads

23 $$\begin{aligned} \Pi (\mathbf {u}, {\mathfrak {d}}) = \underbrace{\int _{{\mathcal {B}} \backslash \Gamma } {\mathfrak {g}}({\mathfrak {d}}) \Psi ( {\varvec{\varepsilon }}_{m}, \varvec{\kappa })\,{\mathrm {d}}\Omega }_{\Pi _{\text {int}}^{b}} + \underbrace{\int _{\Gamma } {\mathcal {G}}_{c} \,{\mathrm {d}}\Gamma }_{\Pi _{\text {fr}}} + \Pi _{\text {ext}}, \end{aligned}$$

where ${\varvec{\varepsilon }}_{m}$ and $\varvec{\kappa }$ represent the membrane- and curvature-related strain resultants.

An alternative formulation to that developed in [32] but also exploiting the Kirchhoff kinematic was proposed by Amiri et al. [33], who employed the the Kirchhoff–Love shell theory and ensured a higher order of continuity through the use of the local maximum-entropy (LME) meshfree approximants. As was therein discussed, the LME technique offered several appealing aspects such as its smoothness, robustness, among other aspects, over alternative meshfree methodologies. Nevertheless, the relying structural shell model led to the use of modified constitutive models (assuming plane stress states) in order to keep the consistency with the kinematic formulation. Regarding the structural concepts, again, Amiri et al. [33] assessed the proposed shell formulation by modeling fracture events in thin and very thin layers, and neglecting the potential occurrence of geometrical nonlinear effects.

More recent shell models including phase field capabilities have been derived within the context of isogeometric discretization schemes. In this setting, Kiendl et al. [34] attained the corresponding extension for brittle and ductile fracture in shells relying on the Kichhoff–Love model, whereas Ambati et al. [35] developed an isogeometric fracture model using the solid shell concept in combination to the assumed natural strain (ANS) method to prevent transverse shear locking effects.

A remarkable contribution in this area was performed by Areias et al. [36], who combined the use of finite strain behavior for both elastic and elasto-plastic constitutive laws with a local remeshing algorithm based on the phase-field values. Moreover, this latter formulation also encompassed the definition of a top and bottom phase field variables within the shell body, as the formulation proposed in “Phase field formulation for solid shells relying on the enhanced assumed strain (EAS) method” section.

Based on the previous discussion it can be seen that, although the shell model herein outlined share some common features with respect to precedent shell models including phase field fracture capabilities, it originally encompassed the development of a locking free shell model as well as the consideration of top and bottom phase field variables in order to capture a nonuniform fracture evolution over the shell thickness. Finally, it is worth mentioning that different modeling aspects from the theoretical and numerical points of view are still open regarding on this matter, which are worth to be investigated.

### Coupling the phase field for brittle fracture and the cohesive zone model for interface debonding

#### General proposed framework

In many engineering application involving heterogeneous media, crack branching and coalescence as well as crack deviations along interfaces can potentially occur. In order to simulate such complex scenarios, in [37], a novel theoretical formulation and its finite element implementation of a new CZM compatible with the phase field model has been proposed. The two models were consistently coupled at the constitutive level, to account at the interface level the effect of damage in the surrounding continuum.

**Fig. 2:**
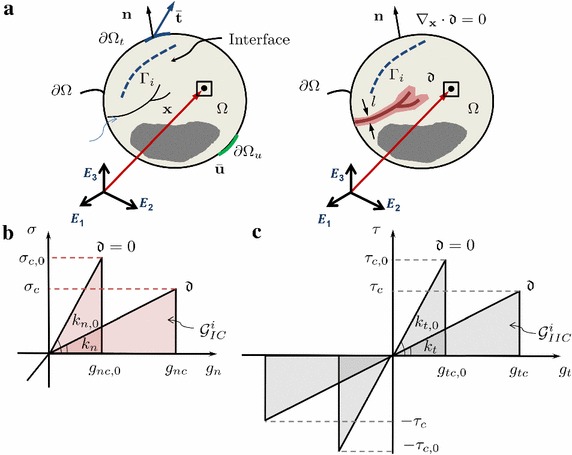
**a** Coexistence between brittle fracture in the bulk and cohesive debonding of an interface within the context of the phase field approach of fracture. Schematic representation of the traction separation law of the CZM which accounts for the phase field variable. **b** Mode I CZM traction $\sigma$ vs. $g_n$. **c** Mode II CZM traction $\tau$ vs. $g_t$

Starting from the vectorial topology defined in “Foundation of the phase field method” section, a generic body with cracks $\Gamma _b$ and prescribed interfaces $\Gamma _i$ is considered (Fig. 2a). The generic point on the interface $\Gamma _{i}$ is denoted by the vector $\mathbf {x}_{c}$. Then, it is recalled the free energy functional which govern the mechanics of the body $\Omega$ defined in Eq. (2):

24 $$\begin{aligned} \Pi (\mathbf {u}, \Gamma ) = \Pi _{\Omega } (\mathbf {u}, \Gamma ) + \Pi _{\Gamma } (\Gamma ) = \int _{\Omega \backslash \Gamma } \psi ^{e}({\varvec{\varepsilon }}) \,\mathrm {d}\Omega + \int _\Gamma {\mathcal {G}}_{c} \,\mathrm {d}\Gamma , \end{aligned}$$

The main idea for the coupling between the phase field model and the CZM is to split the fracture energy function ${\mathcal {G}}_{c}$ into two parts. The first contribution (${\mathcal {G}}_{c}^{b}$) describes the fracture in the bulk and it is modelled with the phase field approach. The second contribution (${\mathcal {G}}^{i}$) attains the cohesive debonding of the interface and it is modelled with a CZM. Then, the free energy functional in Eq. (24) can be rewritten as:

25 $$\begin{aligned} \Pi (\mathbf {u}, \Gamma _{b}, \Gamma _{i}) = \Pi _{\Omega } + \Pi _{\Gamma _b} + \Pi _{\Gamma _i} = \int _{\Omega \backslash \Gamma } \psi ^{e}({\varvec{\varepsilon }}) \,\mathrm {d}\Omega + \int _{\Gamma _{b}} {\mathcal {G}}_{c}^{b} (\mathbf {u},{\mathfrak {d}})\,\mathrm {d}\Gamma + \int _{\Gamma _{i}} {\mathcal {G}}^{i} \left( \mathbf {g}, \mathfrak {h}, {\mathfrak {d}} \right) \,\mathrm {d}\Gamma , \end{aligned}$$

where $\mathbf {g}$ is the displacement discontinuities at the interface, $\mathfrak {h}$ is an history parameter as in [38], and ${\mathfrak {d}}$ is the phase field variable.

The classical linear CZM with tension cut-off [39] was generalized in [37] in order to take into account the effect of the average bulk damage ${\mathfrak {d}}$ of the continuum surrounding the interface flanks. First of all, the cohesive counterpart of the fracture energy in Eq. (25) is decomposed into the sum of the Modes I and II fracture energies, ${\mathcal {G}}_I$ and ${\mathcal {G}}_{II}$, respectively.

In the formulation in [37], the critical opening displacement of the CZM ($g_{c}$) is considered to depend on the bulk damage ${\mathfrak {d}}$ according to the linear relation $g_{c}({\mathfrak {d}})=(1-{\mathfrak {d}})g_{c,0}+{\mathfrak {d}}g_{c,1}$, where $g_{c,0}=g_{c}({\mathfrak {d}}=0)$ and $g_{c,1}=g_{c}({\mathfrak {d}}=1)$. Then, the cohesive traction vs. separation laws for Mode I (and similarly for Mode II) take the form in Fig. 2b, c and are described by the following equations:

26 $$\begin{aligned} \sigma= & {} \left\{ \begin{array}{ll} k_n\dfrac{g_n}{g_{nc}}, &{}\quad \hbox {if } \ 0<\dfrac{g_n}{g_{nc}}< 1; \\ 0, &{}\quad \hbox {if }\ \dfrac{g_n}{g_{nc}}\ge 1, \end{array} \right. \end{aligned}$$

27 $$\begin{aligned} \tau= & {} \left\{ \begin{array}{ll} k_t\dfrac{g_t}{g_{tc}}, &{}\quad \hbox {if } \ 0<\dfrac{g_t}{g_{tc}}< 1; \\ 0, &{}\quad \hbox {if } \ \dfrac{g_t}{g_{tc}}\ge 1. \end{array} \right. \end{aligned}$$

where $\sigma$ and $\tau$ are the Modes I and II tractions, respectively, *g* is the relative displacement, and the subscript *n* and *t* refers to Modes I and II deformation, respectively.

As a result, the apparent stiffness of the cohesive law *k* depends on the damage ${\mathfrak {d}}$ as:

28 $$\begin{aligned} k_n=k_{n,0}\left( \dfrac{g_{nc,0}}{g_{nc}}\right) ^2;\; \, k_t=k_{t,0}\left( \dfrac{g_{tc,0}}{g_{tc}}\right) ^2, \end{aligned}$$

where $k_0$ and $g_0$ are the apparent stiffness and critical relative displacement for ${\mathfrak {d}}=0$.

Finally, without loss of generality, a mixed mode quadratic criterion to couple the modes was introduced to complete the formulation, identifying interface failure:

29 $$\begin{aligned} \left( \dfrac{{\mathcal {G}}_{I}^{i}}{{\mathcal {G}}_{IC}^{i}}\right) ^2 +\left( \dfrac{{\mathcal {G}}_{II}^{i}}{{\mathcal {G}}_{IIC}^{i}}\right) ^2=1, \end{aligned}$$

where ${\mathcal {G}}_{I}^{i}$ and ${\mathcal {G}}_{II}^{i}$ are the dissipated fracture energies which take the form:

30 $$\begin{aligned} {\mathcal {G}}_{I}^{i}({\mathfrak {d}})=\dfrac{1}{2}n_{t,0}g_{n}^2 \dfrac{g_{nc,0}^2}{\left[ (1-{\mathfrak {d}})g_{nc,0}+ {\mathfrak {d}}g_{nc,1}\right] ^2}; \, {\mathcal {G}}_{II}^{i}({\mathfrak {d}})=\dfrac{1}{2}k_{t,0}g_{t}^2 \dfrac{g_{tc,0}^2}{\left[ (1-{\mathfrak {d}})g_{tc,0} +{\mathfrak {d}}g_{tc,1}\right] ^2}. \end{aligned}$$

These dissipated fracture energies are compared in Eq. (29) with the critical fracture energies ${\mathcal {G}}_{IC}^{i}$ and ${\mathcal {G}}_{IIC}^{i}$, which are independent of the phase field damage variable and take the form:

31 $$\begin{aligned} {\mathcal {G}}_{IC}^{i}=\dfrac{1}{2}g_{nc,0}^2k_{n,0}; \, {\mathcal {G}}_{IIC}^{i}=\dfrac{1}{2}g_{tc,0}^2k_{t,0}. \end{aligned}$$

#### Alternative modeling approaches for cohesive- and interface-like phase field fracture formulations: homogeneous and heterogenous media

The modeling framework outlined in the previous section was originally conceived in order to trigger damage events in media with existing interfaces. Nevertheless, in the related literature, other numerical approaches of notable relevance have been proposed in the last few years and whose principal aspects are herein discussed.

Concerning the development of cohesive-like phase field modeling approaches for fracture, Verhoosel and De Borst [40] proposed a seminal technique, whose fundamental motivation concerned the application of the phase field technique to cohesive fracture. For this purpose, the fracture energy can be gradually released and the energy dissipation is governed by the so-called fracture energy function ${\mathcal {G}} = {\mathcal {G}} (\mathbf {g}, \mathfrak {h})$, where $\mathbf {g}$ and $\mathfrak {h}$ denote the displacement gaps across a discontinuity and the history parameter complying with the Kuhn–Tucker conditions. As discussed in [40], this model was basically formulated for triggering fracture events in the bulk. In comparison with the framework described in “General proposed framework” section, the formulation developed in [40, 41] can be conceptually seen as a point of departure for the development of a computational approach which encompasses fracture events in the bulk, based on the brittle phase field approach, and also along an existing interface. Thus, the fracture potential ${\mathcal {G}} = {\mathcal {G}} (\mathbf {g}, \mathfrak {h})$ can be decomposed as follows

32 $$\begin{aligned} \int _\Gamma {\mathcal {G}}_{c} \,\mathrm {d}\Gamma = \int _{\Gamma _{b}} {\mathcal {G}}_{c}^{b} (\mathbf {u},{\mathfrak {d}})\,\mathrm {d}\Gamma + \int _{\Gamma _{i}} {\mathcal {G}}^{i} \left( \mathbf {g}, \mathfrak {h}, {\mathfrak {d}} \right) \,\mathrm {d}\Gamma . \end{aligned}$$

A further extension of the functional outlined in Eq. (32) would concern the combined modeling of cohesive fracture at the bulk and along the existing interfaces. Then, the potential split can be expressed as

33 $$\begin{aligned} \int _\Gamma {\mathcal {G}}_{c} \,\mathrm {d}\Gamma = \int _{\Gamma _{b}} {\mathcal {G}}_{c}^{b} (\mathbf {g}_{b}, \mathfrak {h}_{b},{\mathfrak {d}})\,\mathrm {d}\Gamma + \int _{\Gamma _{i}} {\mathcal {G}}^{i} \left( \mathbf {g}, \mathfrak {h}, {\mathfrak {d}} \right) \,\mathrm {d}\Gamma , \end{aligned}$$

where $\mathbf {g}_{b}$ and $\mathfrak {h}_{b}$ identify the displacement gaps and the history variables in the bulk, respectively. This latter formulation has not been analyzed in the current study, so that the evaluation of its performance is beyond the scope of this paper.

Alternatively, a recent study carried out by Khisamitov and Meshcke [42] introduced a novel variational approach to brittle fracture relying on the use of zero-thickness interface elements. Such a technique is variationally formulated within the spirit of the phase field approach of fracture, whereby a split of the total potential energy of the system into the bulk and interface counterparts was proposed. However, according to [42], in that model fracture events can be only simulated along existing interfaces, representing a notable limitation. In this regard it is worth mentioning that the discrete form of the proposed model [42] was also equipped with an extra nodal degree of freedom in the corresponding FE mesh, which corresponded to the damage variable at the interface. Furthermore, although the authors claimed that it enabled the reduction of the computation cost and the circumvention of the incorporation of the phase field length scale parameter, this technique required the use of nonlocal crack tracking algorithms which can lead to notable implementation tasks for its use in standard or commercial FE packages.

In contrast to the previous numerical techniques and the modeling framework outlined in this paper, Bourdin et al. [43, 44] have investigated the development of fracture events in heterogenous media and thin film-substrate systems. In the former instance [43], fracture phenomena were analyzed based on the variational approach of fracture via a numerical approach denominated as surfing boundary condition and through the exploitation of the elastic heterogeneity concept. The numerical predictions discussed in that study showed patterned fracture events, which strongly depended upon the elastic and fracture mismatch between the constituents. Nevertheless, as the authors stated, they precluded the cases where the interface fracture toughness was different from the bulk values, so that the potential role of the dissipation along the interface was diminished. The second range of applications studied by Bourdin et al. [44] implied thin film-substrate systems under in-plane loadings, whereby similar modeling assumptions as those invoked in [43] were accomplished. In such investigations, the authors distinguished between cracks in the film and debonded surfaces using a unified variational principle, and therefore it can be interpreted as a brittle elastic membrane on a brittle elastic foundation. In any case, in comparison with the current numerical framework (“General proposed framework” section), Bourdin et al. did not consider the potential role of the exiting interfaces in the thin film-substrate systems, which can evolve concomitantly with buckling events [45, 46] and play a crucial role in debonding phenomena in engineering applications.

### Interlaminar and translaminar fracture in laminates combining the 3D approach to brittle fracture for solid shells and a finite elasticity interface finite element for decohesion

**Fig. 3:**
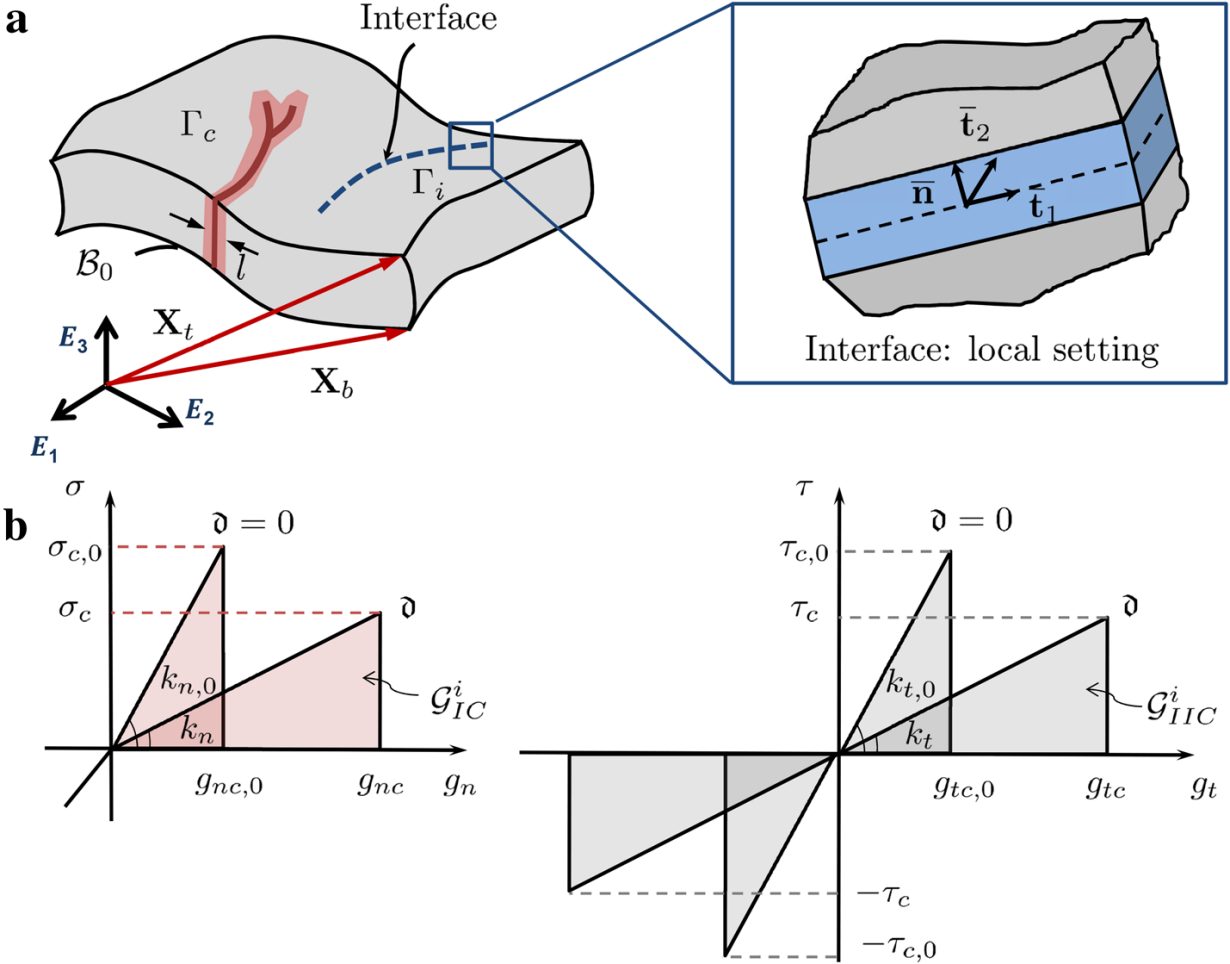
**a** Generic shell body with cracks and prescribed interfaces; **b** traction–separation laws for fracture Modes I and II

The postulation of a 3D interface formulation in the framework of the finite strain was a direct extension of the 2D small strain formulation discussed above. The formulation has been coupled with the solid shell formulation previously presented, see [47]. Considering a generic shell with cracks and a pre-existing interface as shown in Fig. 3a, the dissipative part of the energy functional was split into a bulk contribution and an interface contribution (Eq. 25). This split was introduced in the Hu–Washizu functional (Eq. 8) as:

34 $$\begin{aligned} \Pi (\mathbf {u}, \mathbf {\tilde{E}}, \mathbf {S}, {\mathfrak {d}})&= \int _{{\mathcal {B}}_{0} \backslash \Gamma } {\mathfrak {g}}({\mathfrak {d}}) \Psi ( \mathbf {{E}})\,{\mathrm {d}}\Omega - \int _{{\mathcal {B}}_{0}} \mathbf {S}: \mathbf {\tilde{E}} \,{\mathrm {d}}\Omega \nonumber \\&\quad + \underbrace{\int _{{\mathcal {B}}_{0}} {\mathcal {G}}^{b}_{c} \gamma ({\mathfrak {d}}, \nabla _{\mathbf {X}} {\mathfrak {d}}) \,{\mathrm {d}}\Omega }_{\Pi _{\text {fr}}^{b}} + \underbrace{\int _{\Gamma _{i}} {\mathcal {G}}^{i}_{c}(\mathbf {u},{\mathfrak {d}})\,{\mathrm {d}}\Gamma }_{\Pi _{\text {fr}}^{i}} + \Pi _{\text {ext}}, \end{aligned}$$

where $\Pi _{\text {fr}}^{b}$ is the bulk contribution and $\Pi _{\text {fr}}^{i}$ is the interface contribution.

As compared to the 2D small strain formulation, it is notable to remak that the extension to 3D cases required the introduction of the fracture Mode III within the proposed framework. Then, the constitutive response for a generic fracture mode (Fig. 3b) reads:

35 $$\begin{aligned} S_m=\left\{ \begin{array}{ll} k_m\dfrac{g_m}{g_{m,c}}, &{} \quad \hbox {if }\ 0<\dfrac{g_m}{g_{m,c}}< 1; \qquad \text {with} \quad m = n, t1, t2 \\ 0, &{} \quad \hbox {if } \ \dfrac{g_m}{g_{m,c}}\ge 1, \end{array} \right. \end{aligned}$$

where $S_{m}$ identifies the Piola stress of the interface, being $S_{m,c}$ its corresponding critical value; $k_m$ is the interface stiffness, whereas $g_m$ and $g_{m,c}$ are the displacement gap and its critical value in the local reference system of the interface. The previous cohesive law was further equipped by a penalty formulation in compression with the aim of precluding the material interpenetration at the interface.

Relying on the previous considerations, the fracture energies for a generic fracture mode reads:

36 $$\begin{aligned} {\mathcal {G}}^{i}_{m,c} = \frac{1}{2} S_m g_{m,c} = \frac{1}{2} k_m g_{m,c}^{2}, \qquad \text {with} \quad m = n, t1, t2 \end{aligned}$$

Similarly to the 2D case, the critical relative displacements triggering interface failure encompassed a linear dependence on $\hat{{\mathfrak {d}}}$. On the contrary, the critical energy release rate for each fracture mode was always constant. Then, imposing the independence of the fracture energies for each Mode and the crack phase field value, the following expressions for the energy release rates can be derived:

37 $$\begin{aligned} {\mathcal {G}}_m^{i}({{\mathfrak {d}}})=\dfrac{1}{2}k_{m,0}g_m^2\dfrac{g_{m,c,0}^2}{\left[ (1-{{\mathfrak {d}}})g_{m,c,0}+{{\mathfrak {d}}} g_{m,c,1}\right] ^2} , \qquad \text {with} \quad m = n, t1, t2 \end{aligned}$$

Without loss of generality, a standard quadratic criterion to trigger the interface failure under mixed mode fracture conditions was again adopted as in 2D and render:

38 $$\begin{aligned} \left( \dfrac{{\mathcal {G}}_{n}^{i}}{{\mathcal {G}}_{n,C}^{i}}\right) ^2+\left( \dfrac{{\mathcal {G}}_{t1}^{i}}{{\mathcal {G}}_{t1,C}^{i}}\right) ^2 + \left( \dfrac{{\mathcal {G}}_{t2}^{i}}{{\mathcal {G}}_{t2,C}^{i}}\right) ^2 =1. \end{aligned}$$

Finally, the following tangent constitutive operators at the interface are derived for the subsequent numerical treatment via nonlinear FEM:

39a $$\begin{aligned} \frac{\partial ^{2} {\mathcal {G}}_{c}^{i}}{\partial \mathbf {g}_{\text {loc}}^{2} }&=\left[ \begin{array}{ccc} \hat{\alpha } k_{n} &{} 0 &{} 0 \\ 0 &{} \hat{\beta } k_{t1} &{} 0 \\ 0 &{} 0 &{} \hat{\gamma } k_{t2} \\ \end{array} \right] , \end{aligned}$$

39b $$\begin{aligned} \frac{\partial ^{2} {\mathcal {G}}_{c}^{i}}{\partial \mathbf {g}_{\text {loc}} \partial \hat{{\mathfrak {d}}} }&= \left[ \begin{array}{ccc} g_n k_{n}\dfrac{\partial \hat{\alpha }}{\partial \hat{{\mathfrak {d}}}} &{} g_{t1} k_{t1}\dfrac{\partial \hat{\beta }}{\partial \hat{{\mathfrak {d}}}} &{} g_{t2} k_{t2}\dfrac{\partial \hat{\gamma }}{\partial \hat{{\mathfrak {d}}}} \\ \end{array} \right] , \end{aligned}$$

39c $$\begin{aligned} \frac{\partial ^{2} {\mathcal {G}}_{c}^{i}}{\partial \hat{{\mathfrak {d}}} \partial \mathbf {g}_{\text {loc}} }&=\left[ \begin{array}{c} g_n k_{n}\dfrac{\partial \hat{\alpha }}{\partial \hat{{\mathfrak {d}}}} \\ g_{t1} k_{t1}\dfrac{\partial \hat{\beta }}{\partial \hat{{\mathfrak {d}}}} \\ g_{t2} k_{t2}\dfrac{\partial \hat{\gamma }}{\partial \hat{{\mathfrak {d}}}} \\ \end{array} \right] , \end{aligned}$$

39d $$\begin{aligned} \frac{\partial ^{2} {\mathcal {G}}_{c}^{i}}{\partial \delta \hat{{\mathfrak {d}}}^{2} }&= \dfrac{1}{2}g_{n}^2k_{n}\dfrac{\partial ^2\hat{\alpha }}{\partial \hat{{\mathfrak {d}}}^2}+ \dfrac{1}{2}g_{t1}^2k_{t1}\dfrac{\partial ^2\hat{\beta }}{\partial \hat{{\mathfrak {d}}}^2} + \dfrac{1}{2}g_{t2}^2k_{t2}\dfrac{\partial ^2\hat{\gamma }}{\partial \hat{{\mathfrak {d}}}^2}. \end{aligned}$$

where the terms $\hat{\alpha }$, $\hat{\beta }$ and $\hat{\gamma }$ are given by:

40a $$\begin{aligned} {\hat{\alpha }}&= \dfrac{g_{nc,0}^2}{\left[ (1-{{\mathfrak {d}}})g_{nc,0} + {{\mathfrak {d}}} g_{nc,1}\right] ^2}, \end{aligned}$$

40b $$\begin{aligned} {\hat{\beta }}&= \dfrac{g_{t1c,0}^2}{\left[ (1-{{\mathfrak {d}}})g_{t1c,0} + {{\mathfrak {d}}} g_{t1c,1}\right] ^2} \end{aligned}$$

40c $$\begin{aligned} {\hat{\gamma }}&= \dfrac{g_{t2c,0}^2}{\left[ (1-{{\mathfrak {d}}})g_{t2c,0} + {{\mathfrak {d}}} g_{t2c,1}\right] ^2} . \end{aligned}$$

## Applications to heterogeneous materials and composite structures

In this section we present the numerical applications of the computational models described in the previous section. The first application concerns the simulation of fracture in flat or curved shell structures with in-plane or out-of-plane loading. In the examples, the capabilities of the coupled phase field and CZM formulation are illustrated through various examples concerning heterogeneous materials. In particular, the competition between crack penetration and interface delamination is reproduced and compared with the theoretical results based on linear elastic fracture mechanics (LEFM) predictions. Phenomena such as intergranular/transgranular fracture competition in polycrystalline materials, or interlaminar/translaminar fracture competition in laminates are also outlined.

### Fracture in 3D solid shells

**Fig. 4:**
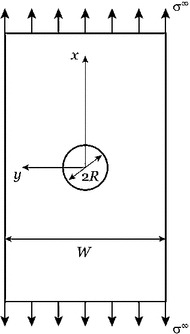
Geometry of the simulated open-hole lamina (from [51])

In the work developed in [48], the capabilities of the phase field approach to fracture, formulated within the finite elasticity solid shell element, have been highlighted in reference to an important technological problem for novel materials to be used in aerospace applications. In particular, failure analysis and strength prediction of an open-hole lamina subjected to tensile loading has been considered (Fig. 4). The problem has been faced using two modelling approaches: the so-called finite fracture mechanics (FFM) method [3], and the phase field model previously described.

Finite fracture mechanics is a theoretical model which rely on a couple energy-stress fracture criterion. It considers the average of the energy release rate and of the stress along a small finite crack path, $l_a$. When the average energy release rate and the average stress overcome their respective critical values, the crack is predict to propagate of the length $l_a$. For details on the FFM approach applied to the present problem, please refer to [48].

These two modelling strategies have been applied for the reproduction of the experimental results of different thin-ply systems performed in [49]. In Table 1, the experimental results and the numerical predictions are compared. For both methods, the relative error is lower than 11$\%$.

The phase field approach results of critical importance and of much easier applicability over FFM when failure analysis of a more complex structure geometry has to be performed. Moreover, the phase field method allows to predict not only the ultimate strength of the material, but also its stiffness evolution along the loading process.

**Fig. 5:**
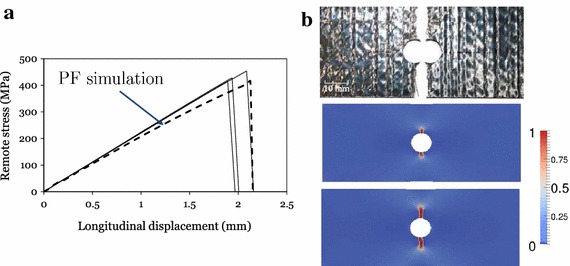
**a** Experimental and numerical remote stress vs. displacement curves; **b** experimental–numerical crack path for open-hole specimens

Figure 5a shows the comparison between the experimental results and numerical predictions in the remote stress vs. displacement curves for the specimen T700/AR-2527. The behaviour of the material is strongly brittle, exhibiting a quasi perfect linear evolution upon abrupt failure. This behaviour was qualitatively reproduced by the phase field prediction. The crack path was also in good agreement with the experimental one (Fig. 5b). However, the stiffness of the laminate was slightly underestimated. This discrepancy can be probably addressed to the fact that the laminate was considered as an unique homogeneous body instead of the sum of different orthotropic laminae.

**Table 1:** Experimental results and numerical predictions

Composite system and specimen geometry	Experimental results (MPa)	Predictions		Relative error	
		FFM (MPa)	PF (MPa)	FFM (%)	PF (%)
T700/AR-2527—laminate 1					
W/(2R) = 4; 2R = 3 mm	432	465	415	7.8	− 3.9
W/(2R) = 4; 2R = 6 mm	385	392	368	1.9	− 4.4
W/(2R) = 4; 2R = 10 mm	367	342	361	− 5.8	− 1.6
T700/AR-2527—laminate 2					
W/(2R) = 4; 2R = 3 mm	448	447	403	− 0.3	− 10.4
W/(2R) = 4; 2R = 6 mm	390	385	358	− 1.2	− 8.2
W/(2R) = 4; 2R = 10 mm	380	338	345	− 11.1	− 9.2
M40JB/ThinPreg 80EP/CF					
W/(2R) = 6; 2R = 1 mm	551	559	524	1.5	− 6.3
W/(2R) = 6; 2R = 2 mm	470	475	447	1.0	− 5.9
W/(2R) = 6; 2R = 6 mm	365	356	351	− 2.5	− 3.8

Columns 1, 2 show the specimens geometries for each type of composite material. Column 3 shows the experimental failure stress of the specimen. Column 4, 5 show the numerical prediction of the finite fracture mechanics method and the phase field method. Columns 6, 7 show the errors of the predictions with respect to the experimental results

**Fig. 6:**
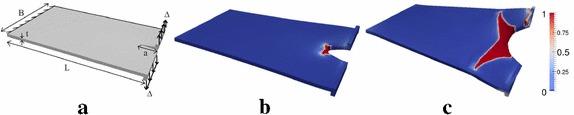
Tearing test. **a** Geometry and loading condition of the test; **b** crack propagation; **c** specimen failure (from [24])

**Fig. 7:**
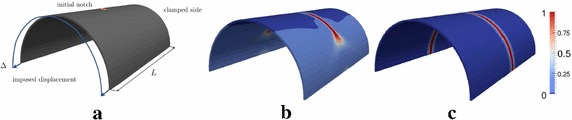
Tensile test of a cylindrical shell. **a** Geometry and loading condition of the test; **b** crack propagation; **c** specimen failure (from [24])

More applications of the finite elasticity solid shell element have been presented in [24]. The three-dimensional formulation has been used in order to reproduce the fracture of a notched plate under out-of-plane loading (Fig. 6a) and the fracture of a cylinder under tension loading (Fig. 7a). In the former case, the loading condition triggered Mode III fracture (Fig. 6b). Subsequently, all the three fracture Modes became activated and the fracture path was deviated from the initial straight direction (Fig. 6c).

In the cylindrical shell application, it is shown the ability of the model to simulate crack propagation in curved geometries. Figure 7b, c shows how the crack perfectly followed the Mode I crack direction along the cylindrical shape.

### The problem of the crack impinging on an interface studied using the phase field approach to fracture coupled with the cohesive zone model

The modelling framework previously described has been used for the study of crack propagation in heterogeneous materials. In such cases, the interaction between crack propagation in the bulk and the delamination along an interface is a very challenging task and it is still an open issue from the point of view of fundamental research. When a crack impinges on an interface, various scenarios can take place [37]: (i) crack penetration, (ii) crack deflection or (iii) crack branching. The first situation refers to the case when the crack crosses the interface without development of delamination. In the second case, delamination occurs and the crack deflects. Finally, the last case comprehends the penetration of the crack with possible development of crack branches after crossing the interface.

**Fig. 8:**
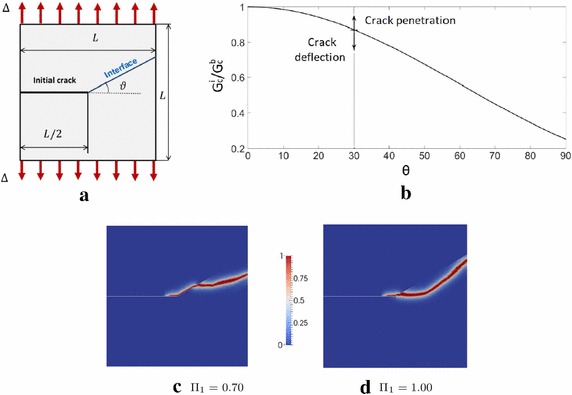
**a** Geometry considered to study the effect of a crack impinging on an interface; **b** curve which separate the crack penetration and deflection cases for different impinging angles; transition from crack deflection, **c** to crack penetration, **d** for a brittle interface $(\Pi _2\rightarrow 0)$ with angle $\theta = 30^\circ$ (from [37])

In [37], the authors reproduced these fracture modes in the system in Fig. 8a. They made use of the novel interface finite element formulation compatible with the phase field. This numerical approach has been supported by a dimensional analysis which shade light on the variables governing the problem. The following dimensionless numbers were defined:

41 $$\begin{aligned} \Pi _1 = \dfrac{{\mathcal {G}}_{\text {c}}^{\text {b}}}{{\mathcal {G}}_{\text {c}}^{\text {i}}} \qquad \qquad \Pi _2 = \dfrac{{\mathcal {G}}_{\text {c}}^{\text {i}} E}{\sigma _{\text {c}}^2 L} \end{aligned}$$

where ${\mathcal {G}}_{\text {c}}^{\text {b}}$ and ${\mathcal {G}}_{\text {c}}^{\text {i}}$ stand for the fracture energies of the bulk and of the interface, respectively, $\sigma _{\text {c}}$ is the apparent strength of the material, *E* is its Young modulus, and *L* is the specimen size (Fig. 8a).

For the considered system, the possible fracture modes are crack penetration or deflection (Fig. 8b). Dimensional analysis suggests that $\Pi _2$ rules the size-scale effect given by the cohesive interface, and it is in fact proportional to the ratio $l_{\mathrm {CZM}}/L$ [37]. When $\Pi _2\longrightarrow 0$, the interface was set very brittle and LEFM predictions were recovered. The dimensionless number $\Pi _1$, on the other hand, governed the competition between crack penetration and deflection, as shown in Fig. 8b. On the contrary, as $\Pi _2$ increases, more relevant cohesive phenomena at the interface are developed, setting a competition between these two parameters which rules the mechanical system. Figure 8c, d shows the results of the simulations of different values of the ratio $\Pi _1$ obtained through the computational model in [37].

**Fig. 9:**
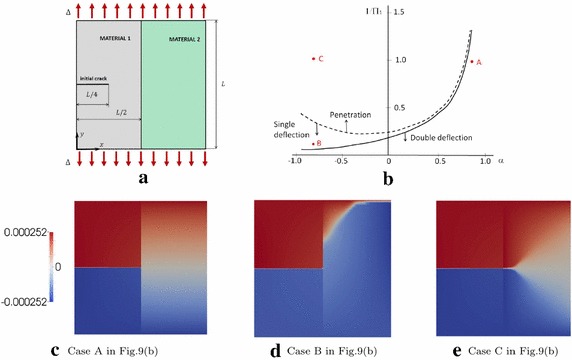
**a** Geometry of the tensile test of a bi-material system; **b** curve which separate the crack penetration, crack single deflection and crack double deflection cases; transition from double deflection (**c**) to single deflection (**d**) and crack penetration (**e**) for a brittle interface $(\Pi _2\rightarrow 0)$ varying the ratio $1/\Pi _1$. The contour plots scale is a dimensional vertical displacement (from [37])

The behaviour discussed above changed when we have a bi-material system like in Fig. 9a. In this case, the Dundurs’ parameters $\alpha$ and $\beta$ [50] should be considered. Based on the value of the ratio $1/\Pi _1$, three different crack patterns can be simulated depending on the LEFM-predicted curves shown in Fig. 9b and separating double deflection from single deflection and penetration failure modes. The numerical predictions whose crack patterns are shown in Fig. 9c–e, were in very good agreement with LEFM predictions.

### Fracture in anisotropic polycrystalline silicon

Another application of the computational model based on the phase field model and the compatible interface finite element can be found in [51]. In this work, the competition between intergranular and transgranular fracture in polycrystalline materials has been studied, considering anisotropic constitutive relations for the grains, depending on their crystallographic orientation. The phase field model has been used to simulate transgranular fracture, while cohesive interface finite elements have been used to depict intergranular fracture.

Here, the phase field formulation of the potential energy presents the follows expression:

42 $$\begin{aligned} \Pi (\mathbf {u}, {\mathfrak {d}}) = \int _{\Omega } \psi ({\varvec{\varepsilon }} ({\varvec{C}}^{\langle abc\rangle }),{\mathfrak {d}})\,\mathrm {d}\Omega +\int _\Omega {\mathcal {G}}^{\langle abc\rangle } \gamma ({\mathfrak {d}}, \nabla _{\mathbf {x}} {\mathfrak {d}})\,\mathrm {d}\Omega , \end{aligned}$$

where ${\varvec{C}}^{\langle abc\rangle }$ is the constitutive matrix corresponding to a grain with crystal orientation $\langle abc\rangle$ and ${\mathcal {G}}^{\langle abc\rangle }$ is the corresponding fracture energy.

**Fig. 10:**
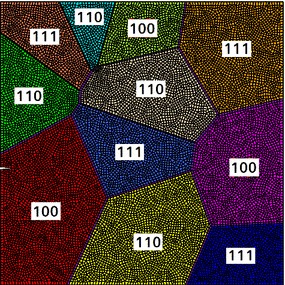
Finite element mesh of the polycrystalline silicon photovoltaic cell. The specimen has a notch on the left edge. The grain orientations are specified by the Miller indices in the grains and by the different colours (from [51])

**Fig. 11:**
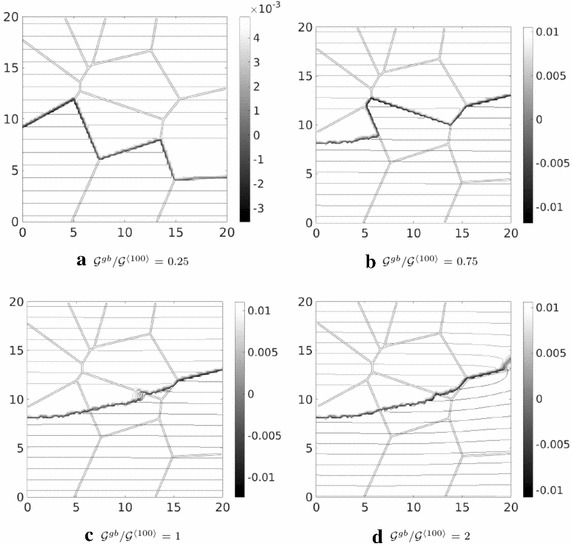
Contour plots of inelastic displacement field, illustrating the crack path for different values of ${\mathcal {G}}^{gb}/{\mathcal {G}}^{\langle 100\rangle }$ (from [51])

This computational model has been applied to the description of fracture of polycrystalline silicon solar cells, see an example of finite element mesh in Fig. 10. Silicon grains were connected to each other by means of zero-thickness interface finite elements. The square domain in Fig. 10 presented an edge notch on the left side. A tensile test has been simulated by considering different values of the ratio between the grain boundary fracture energy and the fracture energy of the grain with orientation $\langle 100 \rangle$, i.e., ${\mathcal {G}}^{gb}/{\mathcal {G}}^{\langle 100\rangle }$. The simulations showed that for low values of ${\mathcal {G}}^{gb}/{\mathcal {G}}^{\langle 100\rangle }$, failure was mostly the result of intergranular fractures (Fig. 11a). By increasing the value of the ratio ${\mathcal {G}}^{gb}/{\mathcal {G}}^{\langle 100\rangle }$, transgranular fracture developed from the notch and it competed with intergranular debonding (Fig. 11b). For much higher high values of ${\mathcal {G}}^{gb}/{\mathcal {G}}^{\langle 100\rangle }$ greater than 1, failure was mostly dominated by transgranular fracture (Fig. 11c, d).

### Fracture and delamination in 3D laminates

The competition between fracture and delamination in 3D composite laminates has been deeply investigated in [47] within a finite elasticity framework. The model was based on the phase field formulation within the solid shell element and of the 3D interface elements for large relative displacements.

**Fig. 12:**
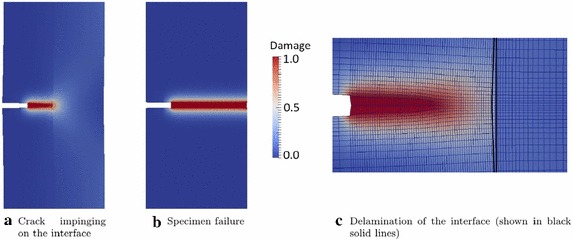
**a**, **b** Two steps of crack propagation; **c** close-up view of delamination when the crack is approaching the interface (from [47])

**Fig. 13:**
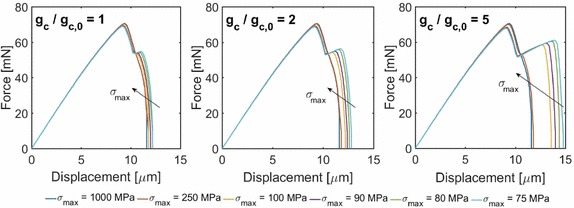
Force displacement curve for different $\sigma _{\text {max}}$ and different ratio $g_{\text {c}} / g_{\text {c,0}}$ (from [47])

The influence of the constitutive coupling between phase field and the CZM has been a primary topic of investigation. To this aim, a single-edge notched specimen with an interface has been numerically tested under tensile load (Fig. 12). A parametric analysis has been performed by varying the interface strength ($\sigma _{\max }$) and the ratio between the initial (${\mathfrak {d}} = 0$) and the final (${\mathfrak {d}} = 1$) critical crack openings. The predicted crack pattern based on the model parameters used was characterized by the following features (Fig. 12): the crack was predicted to penetrate the interface and then it propagated in pure Mode I, without crack deflection or branching, although partial decohesion of the interface takes place. Coupling between the CZM and damage in the surrounding continuum led to different post-peak branches in the force-displacement curves depending on the ratio between the critical opening displacements for the undamaged and fully damages states, $g_c/g_{c,0}$, see Fig. 13. When debonding was estimated to occur, two drops in the load carrying capacity of the mechanical system were observed: the first one corresponds to the complete failure of the left part of the specimen, and the second one is associated with the failure of the right part. The amount of energy dissipated in this post-peak evolution depends on the amount of delamination, which is an increasing function of the ratio $g_c/g_{c,0}$.

**Fig. 14:**
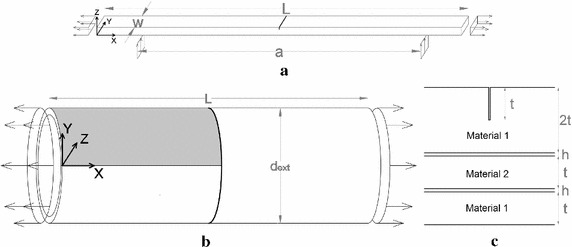
Geometry of the 4-point-bending specimen in **a** and geometry of the cylinder under tension in **b**. Composite composition through the thickness in **c** (from [47])

**Fig. 15:**
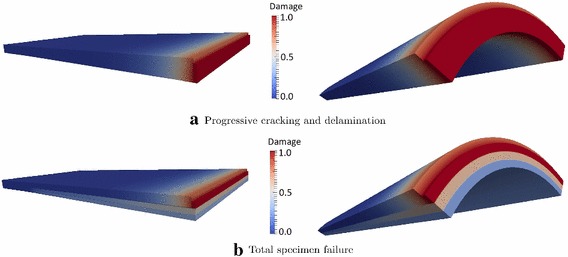
Crack propagation and delamination in a notched sandwich panel. **a** Flat geometry under tensile and 4-point banding loading; **b** cylindrical geometry under tensile loading (from [47])

**Fig. 16:**
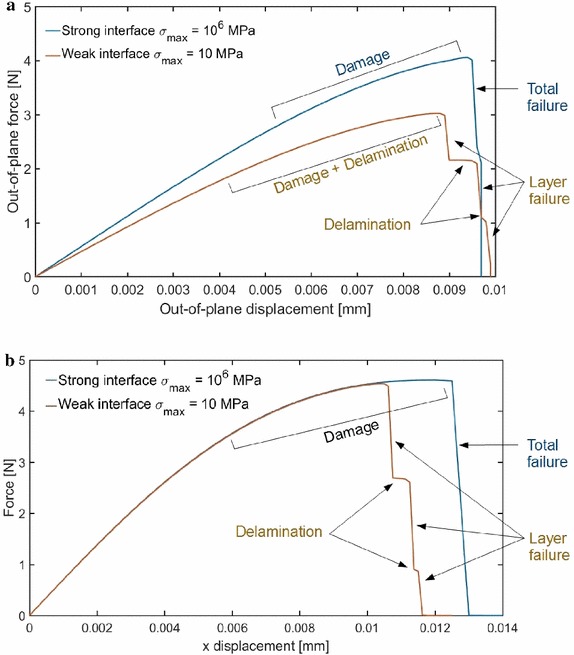
Force-displacement curves. **a** Sandwich panel under tension and 4-point-bending, **b** Sandwich cylinder under tension (from [47])

This competition between crack propagation and delamination has been also captured in laminate systems. In this case, the crack propagates through the thickness of the solid shell and delamination occurs at the interface between two laminae, as in the sandwich panel with a notch in the middle of the span that propagates over the complete width of the topmost layer (Fig. 14a). The panel was subjected to in-plane tensile forces and out of plane displacements, to simulate a 4-point bending test. A sandwich panel with the same internal structure but with cylindrical shape has also been tested, see Fig. 14c and with Fig. 14b. This specimen was subjected to a tensile load. In both simulations, depending on the tensile strength of the CZM, delamination and crack propagations were estimated to take place simultaneously. Figure 15 shows the results of the two simulations in which the crack propagates through the laminae and delamination also occurs. Again, delamination events are fully captured by the force-displacement curve as shown in Fig. 16. Moreover, in line with previous considerations, delamination causes a delay of the crack in propagating from a layer to another.

**Fig. 17:**
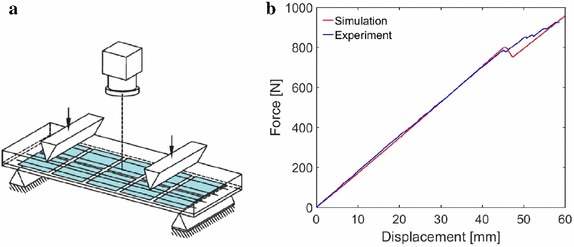
4-point bending test of a photovoltaic panel. **a** Experimental test setup, **b** force displacement curve (from [47])

Finally, an industrial application of this computational model has been proposed in [47] in relation to a 4-point bending test of a photovoltaic module (Fig. 17a). In detail, the module has been discretized as a composite made of 5 layers in which the middle layer is a brittle silicon solar cell, encapsulated by an adhesive material made of EVA, while the topmost and bottommost layers are made of glass and a polymeric material, respectively. The experimental 4-point bending test performed in [52] shows the development of microcracks in the silicon layer. This phenomenon has been captured by the present computational model in terms of overall force-displacement curve (Fig. 17b). The only difference, which requires further developments, regards the fact that the silicon layer is predicted to fail as a result of a single crack in the mid-span position, rather than as a result of multiple microcracks probably due to material defects which are not modelled in the present approach.

### Micromechanics of fibre reinforced composite materials

This application illustrates the prediction of crack initiation and evolution in fibre reinforced composite materials from a micromechanical perspective, so that the different constituents are defined according to their respective mechanical and fracture properties. This application has received a great of attention in the scientific community due to the fact that micromechanical analysis can shed light to macromechanical failure mechanisms in composite materials, especially those associated with matrix-dominated failure [53–55]. In this context, the main capabilities of the proposed numerical framework, differing from alternative techniques, is the fact that a direct competition between fibre–matrix debonding along the existing interface and matrix failure can be carried out throughout the numerical analysis.

The numerical experiment under consideration concerned with the problem of fiber–matrix debonding caused by transverse loads under the assumption of plane strain conditions. Therefore, the problem geometry was reduced to the plane perpendicular to the fiber axis, which was defined by a circular inclusion with radius $$R = 0.01251$$ mm. The lateral size of the square domain was set equal to $$ L = 0.1$$ mm. Similarly previous investigations [53], both matrix and fiber were assumed to be linear elastic materials, and the fibre–matrix interface was assumed perfectly bonded. Material properties for the fibre and matrix are reported in Table 2, whereas the interface properties are listed in Table 3 [56, 57]. We also exploited the symmetry of the problem so that only one quarter of the domain was considered for the current simulation. Note however that, a more comprehensive analysis of the different aspects that govern the current application is beyond the scope of the present paper, and will be discussed in a forthcoming investigation.

**Table 2:** Mechanical properties: fibre and matrix

Material	*E* (GPa)	$\nu$	${\mathcal {G}}_c$ (N/mm)	*l* ($\upmu$m)
Fibre	210	0.22	16	0.45
Matrix	2.8	0.33	0.016	0.45

**Table 3:** Mechanical properties of the fibre–matrix interface

Interface property	$\sigma _{C}$ (MPa)	${\mathcal {G}}_{C}$ (N/mm)
Fracture Mode I	75	0.002
Fracture Mode II	90	0.008

**Fig. 18:**
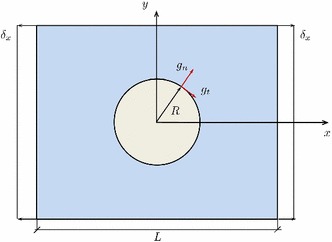
Micromechanics of fibre reinforced composite materials: geometry and boundary conditions

The domain was discretized with first-order 30,274 elements for the matrix, 1200 elements for the interface and 19,095 for the fibre. The simulations were conducted under displacement control, so that a prescribed displacement $\delta _x$ (the imposed strain reads $\tilde{\varepsilon } = \delta _{x}/L$) was imposed at the lateral edges of the domain, see Fig 18. The fiber–matrix interface failure was modeled by the proposed tension cut-off interface response, whose apparent stiffness was coupled with the evolution of the phase field fracture variable of the surrounding bulk.

**Fig. 19:**
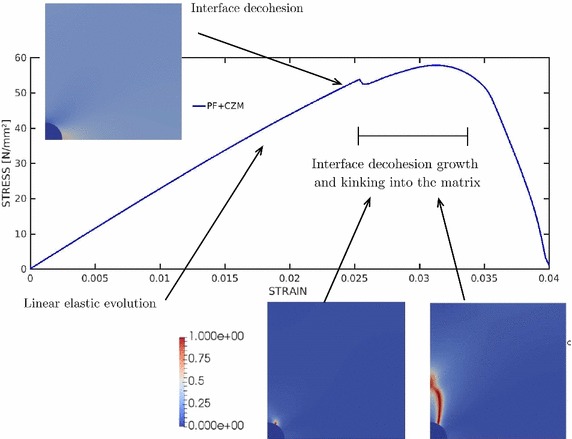
Micromechanics of fibre reinforced composite materials: stress–strain evolution curve and damage pattern at different stages of the simulation

**Fig. 20:**
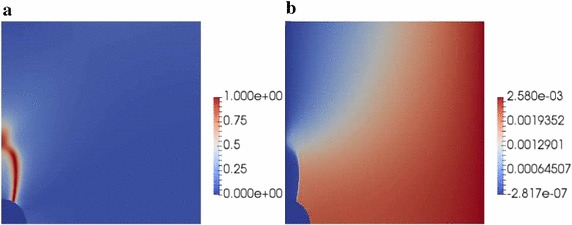
Micromechanics of fibre reinforced composite materials: **a** final damage pattern. **b** Final horizontal displacement

The corresponding load-displacement evolution curve of the current analysis is shown in Fig 19. In this graph, it can be observed that the system response was governed by three different phases:

- The first stage of the simulation displayed a linear elastic evolution of the system prior damage initiation.
- In line with [53, 55], failure of the system was initially ruled by the fibre–matrix decohesion along the interface up to around an angle of around 45$^\circ$. Damage onset and progression along the interface was captured by the sudden drop in the evolution curve, featuring a snap-through instability. Note that this prediction presents remarkable differences with that discussed in [54] whereby only matrix failure along the numerical study was reported. Nevertheless, the abrupt character of damage occurrence reported [54] in was also captured by the current modeling framework.
- The third stage of the curve predicted the subsequent interface damage and the posterior kinking into the matrix. The first phase of damage growth within this range was characterized by a interface failure growth. Afterwards the interface crack was predicted to kink into the adjacent matrix experiencing a stable growth since the stress–strain evolution curve displays a continuous increase along the loading path. Note that the kinking angle for the current configuration was predicted to occur at around 62$^\circ$, which satisfactorily agreed with the experimental observations reported in [53, 55] and the references given therein.

Finally, the final damage pattern and the horizontal displacement field of the system is shown in Fig. 20, whereby the arising discontinuity due to the crack occurrence can be clearly appreciated.

## Conclusions

The phase field model for brittle fracture has been largely used in the last few years and it has seen many developments in order to provide a tool for quantitative fracture mechanics simulations. In this review article, some advancements to simulate complex fracture phenomena in heterogeneous materials and composites, motivated by the ERC Starting Grant CA2PVM project (http://musam.imtlucca.it/CA2PVM.html), have been presented.

The integration of the phase field model in a 3D finite elasticity formulation increased the range of applications of the model. Three-dimensional flat or curved surfaces with in-plane or out-of-plane loading can now be easily simulated. Another important advancement has regarded the coupling of the phase field approach with the cohesive zone model. This progress allows the simulation of heterogeneous structures and materials like composites or polycrystals. The fundamental aspects of the proposed modeling framework were also compared with alternative formulations, pinpointing the common and differentiating underlying hypotheses.

From the quantitative standpoint, the proposed approaches represent an accurate tool to simulate fracture phenomena such as intergranular/transgranular or interlaminar/translaminar fractures, reproducing complex crack paths typical of heterogeneous materials in 2D and in 3D. Finally, preliminary results regarding the micromechanical failure of fibre reinforced composite haven been also addressed, which satisfactorily agreed with previous numerical studies and experimental observations.

## Abbreviations

FP7: framework program 7
ERC: European Research Council
CA2PVM: Multi-field and Multi-scale Computational Approach to Design and Durability of PhotoVoltaic Modules
FEM: finite element method
XFEM: extended finite element method
FFM: finite fracture mechanics
EFEM: embedded finite element method
CZM: cohesive zone model
EAS: enhanced assumed strain
LEFM: linear elastic fracture mechanics
EVA: ethylene-vinyl acetate
